# Fear of large carnivores is tied to ungulate habitat use: evidence from a bifactorial experiment

**DOI:** 10.1038/s41598-021-92469-5

**Published:** 2021-06-21

**Authors:** Haley K. Epperly, Michael Clinchy, Liana Y. Zanette, Robert A. McCleery

**Affiliations:** 1 Department of Wildlife Ecology and Conservation, School of Natural Resources and the Environment, University of Florida, Gainesville, FL 32611 USA; 2 Department of Biology, Western University, London, ON N6A 5B7 Canada; 3 University of Florida, 110 Newins-Ziegler Hall, PO Box 110430, Gainesville, FL 32611-0430 USA

**Keywords:** Ecology, Behavioural ecology, Community ecology, Conservation biology

## Abstract

The fear large carnivores inspire in large ungulates has been argued to have cascading effects down food webs. However, a direct link between ungulate habitat use and their fear of large carnivores has not been experimentally tested. To fill this critical gap, we conducted a bi-factorial experiment in an African savanna. We removed shrub cover and broadcast large carnivore vocalizations (leopard, hyena, dog) or non-threatening control vocalizations in both experimentally cleared and shrubby control sites. We recorded the proactive (frequency of visitation) and reactive (fleeing or vigilance) responses of multiple prey (impala, warthog, nyala and bushbuck). Critically, we found a significant proactive–reactive interaction. Ungulates were 47% more likely to run after hearing a predator vocalization in shrubby control sites than experimental clearings, demonstrating that ungulates perceived less fear from large carnivores in open habitat (clearings). Consistent with this finding, ungulates visited clearings 2.4 times more often than shrubby control sites and visited shrubby control sites less often at night, when large carnivores are most active. Combined with results from previous experiments demonstrating that the disproportionate use of available habitats by large ungulates can alter ecosystem properties, our experiment provides critical evidence that the fear large carnivores inspire in large ungulates can cause trophic cascades.

## Introduction

Globally, landscapes are increasingly devoid of large carnivores, often due to human-large carnivore conflict^1^. The loss of these apex predators has often coincided with considerable alterations in community composition at multiple lower trophic levels^1,2^. Attributing these large-scale environmental changes to large carnivore declines is controversial when considering whether the fear large carnivores inspire in large herbivores and mesocarnivores can contribute to causing trophic cascades^3–5^. Theory^6,7^ and manipulations involving relatively small species conducted mainly in micro- or meso-cosms indicate that fear can induce trophic cascades^8,9^. However, given the importance to conservation and human-large carnivore conflict, there have been justifiable calls for more evidence linking the fear large carnivores inspire to the behavioral changes in prey that can cause trophic cascades^3–5^. Specifically, key gaps remain in the experimental evidence concerning cascades caused by the fear large carnivores inspire in large ungulates. Understanding each step of a fear-induced cascade is often constrained by the difficulty of manipulating fear and environmental features on scales relevant to free-living wildlife, and the difficulty of scale is compounded when the apex predator and their prey are large mammals^3,5,10^. Nonetheless, to understand how large mammals can initiate trophic cascades^11^, there is a critical need to simultaneously manipulate both fear and habitat features.

Experiments on large ungulates, primarily involving fenced exclosures, have clearly demonstrated that reducing large ungulate use of certain areas can cause marked changes in the abundance and species richness of vegetation, particularly woody plants^5,12–14^. Field experiments manipulating vegetation cover have also demonstrated that ungulates’ preferential use of open habitats cannot be explained by foraging resources^13^. It follows that if fear of large carnivores shapes habitat use by large ungulates, cascading effects on the vegetation in these areas should occur^3,13,15^. To experimentally demonstrate that fear of large carnivores is a causal mechanism mediating large ungulate habitat use, and thus a driver of cascading effects, it is necessary to manipulate both: 1) habitat characteristics (e.g., vegetation structure) to verify that ungulates use or avoid areas with these characteristics; and 2) fear (perceived predation risk) to establish that these same ungulates react less fearfully to large carnivores in frequented habitat and more fearfully in avoided habitat^3^. The handful of experiments to date addressing trophic cascades have manipulated habitat characteristics^13,16,17^ or fear^5^ but not both simultaneously. The critical gap in our understanding of trophic cascades concerns the interaction between fear and habitat features, which can only be comprehensively evaluated through a bi-factorial experiment manipulating both^3^.

Just as there have been rapid global declines in large carnivores in recent decades, there have similarly been rapid global shifts in vegetation structure^18^. African savannas, for example, are losing large carnivores^1^ and witnessing the broadscale replacement of grasses with woody vegetation^19^. This creates extensive woody patches that are avoided by many of the region’s mid-sized ungulate grazers and browsers^17,20^.

While increased woody vegetation appears to alter ungulates perceptions of fear and responses to predators^5,13,17^, these perceptions may be mediated by the time of day, potentially increasing during times of increased predator activity^13,21^. Prey’s fear response may also be mediated by predator species^22,23^, with more lethal predators generating greater responses^24,25^. There is a growing need to consider a more complete range of behaviors (e.g., ‘*reactive*’ and ‘*proactive*’ behaviors) to better understand prey species responses to complex systems with multiple predators^10,15,26^. Furthermore, research to date has rarely considered the potential for interactive proactive–reactive responses, whereby ungulates show a greater reactive response in areas with dense shrubby vegetation due to the heightened predation risk^26^, and this remains to be experimentally tested.

The overarching goal of this study was to fill the gaps in our current understanding of how the fear large carnivores inspire in large ungulates may contribute to causing trophic cascades. To accomplish this goal we conducted a bi-factorial experiment in which we manipulated both habitat characteristics (by reducing shrub cover) and fear (by broadcasting large carnivore vocalizations). Measuring both proactive and reactive antipredator responses, we evaluated how ungulate species in an African savanna responded to large carnivore vocalizations (leopard [*Panthera pardus*], hyena [*Crocuta crocuta*], dog [*Canis lupus familiaris*]), or non-threatening control vocalizations (bird calls), in both experimentally cleared and shrubby control sites. We predicted that (1) compared to their responses in experimental clearings, ungulates would react more fearfully (e.g., running) to large carnivore vocalizations in shrubby control areas due to decreased visibility and lack of escape routes^13,16,17,26,27^; (2) consistent with experimental clearings being less fear inducing, we expected ungulates to use them more often^13,16,17^; (3) particularly at night when their predators are more likely to be active^13,28,29^; and (4) ungulates would react more fearfully when exposed to the vocalizations of more lethal predators (i.e., leopard)^26,30^.

## Results

Using an Automated Behavioral Response (ABR) system, which begins recording video once motion is detected and then broadcasts a carnivore or control (bird) vocalization 3-s. after motion is detected, we recorded 850 videos of ungulates over 288 camera-trap nights. We classified 358 videos as independent first exposures, of which 33 were from our naturally open site. We recorded 12 species, the 4 most common species being: impala (*Aepyceros melampus*; n = 110 first exposure videos), nyala (*Tragelaphus angasii*; n = 81), bushbuck (*Tragelaphus scriptus*; n = 51) and warthog (*Phacochoerus africanus*; n = 34). We found no difference in vigilance behavior before the initiation of treatment vocalizations (large carnivores x̄ = 0.95 s., SE = 0.12 s., controls x̄ = 0.92 s., SE = 0.14 s.). For animals that did not run, we recorded an average of 22.4 s. of observation time after the vocalization started.

### Reactive responses to experimental predator vocalizations

**Figure 1:**
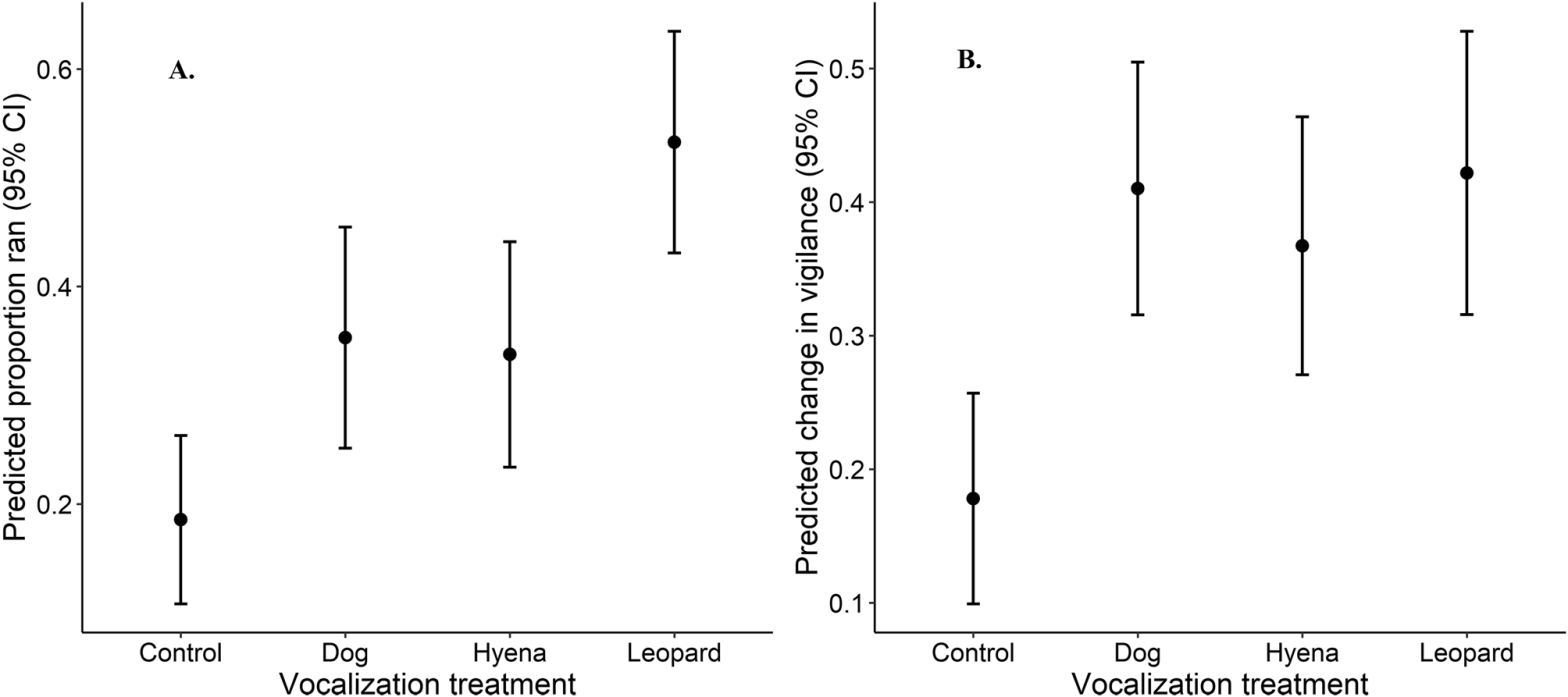
Predicted responses and 95% CIs from generalized linear models of ungulates running (**A**) or changing proportion of time vigilant (**B**) after hearing vocalizations of controls (birds), dogs, hyenas and leopards.

We evaluated the reactive responses of all ungulates (pooled) and common ungulate species to large carnivore vocalizations using generalized-linear models (glms) with running (*Ran*) and vigilance behavior (*Vigilance*) as response variables. All ungulates (pooled) demonstrated significant reactive responses to the vocalizations of each of the 3 large carnivore species (leopard, spotted hyena, and dog). Ungulates were significantly more likely to run after each large carnivore species’ vocalizations compared with controls (Fig. 1A; dog: β = 0.87 ± 0.35 SE p = 0.012; hyena: β = 0.80 ± 0.35 SE p = 0.022; leopard: β = 1.61 ± 0.33 SE p < 0.001). Hearing leopards evoked a significantly stronger response than either dogs or hyenas, with ungulates (pooled) being 50% more likely to run from leopard vocalizations than either dog (β = -0.74 ± 0.31 SE p = 0.017) or hyena (β = -0.81 ± 0.32 SE p = 0.011). Ungulates that did not run were also significantly more vigilant after hearing each type of large carnivore vocalization compared with controls (Fig. 1B; dog: β = 0.23 ± 0.06 SE p < 0.001; hyena: β = 0.19 ± 0.06 SE p < 0.001; leopard: β = 0.24 ± 0.07 SE p < 0.001). In contrast to running, ungulates were not significantly more vigilant after hearing leopard vocalizations compared with hyena or dog.

**Figure 2:**
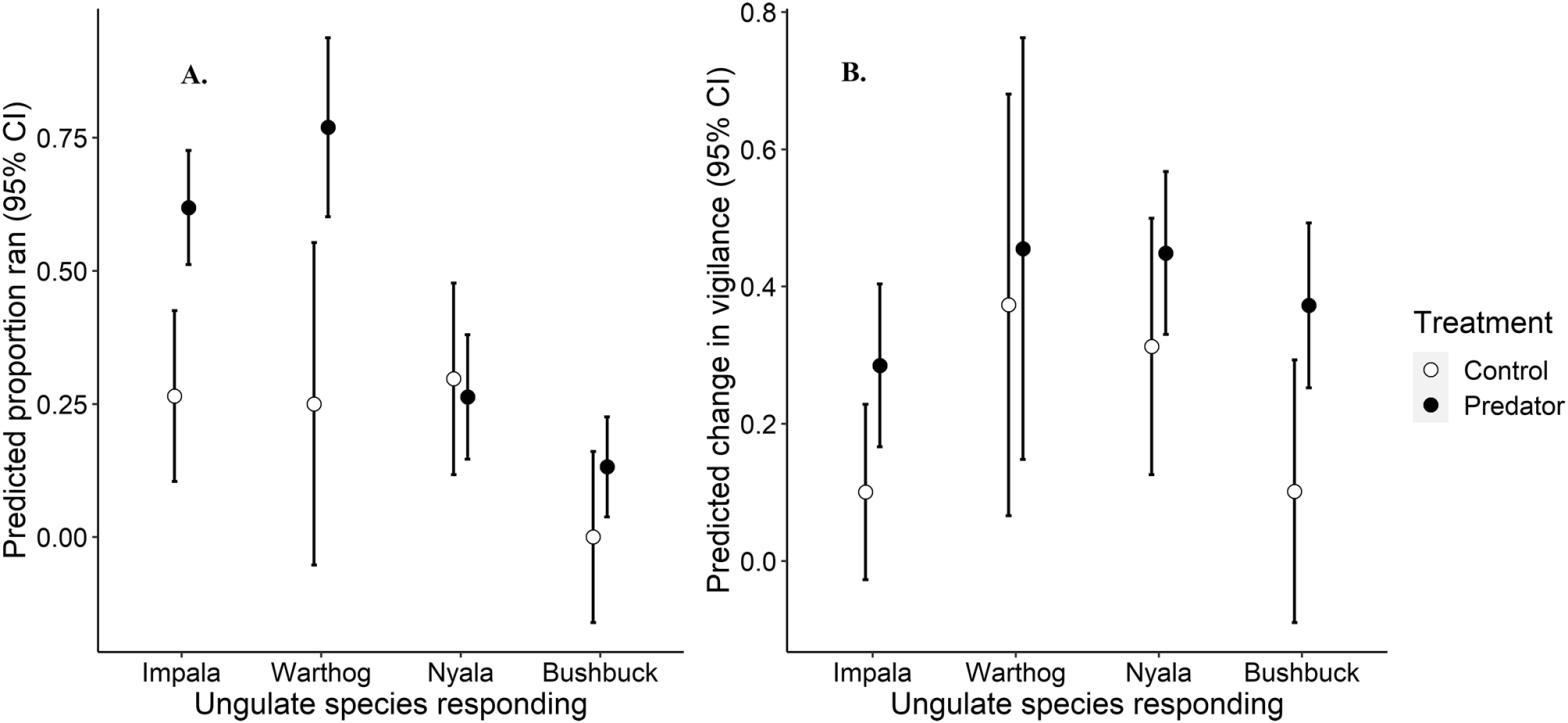
Predicted responses and 95% CIs from generalized linear models of the four most frequently detected ungulate species (impala, warthog, nyala and bushbuck) running (**A**) or changing proportion of time vigilant (**B**) after hearing vocalizations of controls (birds) and large carnivores (dogs, hyenas and leopards).

Three of the four common ungulate species demonstrated significant reactive responses after hearing large carnivore vocalizations, but they differed in the strength and nature of their responses. Both impala and warthogs were significantly more likely to run (impala: β = 0.35 ± 0.10 SE p < 0.001; warthog: β = 0.52 ± 0.18 SE p = 0.006) to large carnivore than control vocalizations (Fig. 2A). Impala were 2.4 times more likely to run after hearing large carnivore compared to control vocalizations, and warthogs were 3.1 times more likely. Nyala and bushbuck, in contrast, were not significantly more likely to run after hearing large carnivore compared to control vocalizations. Considering individuals that did not run, all 4 species showed the same pattern of being more vigilant after hearing large carnivore than control vocalizations (Fig. 2B), with this pattern being significant for both impala and bushbuck (impala: β = 0.18 ± 0.09 SE p = 0.043; bushbuck: β = 0.27 ± 0.12 SE p = 0.023).

### Interactive proactive–reactive responses to the bi-factorial experiment

**Figure 3:**
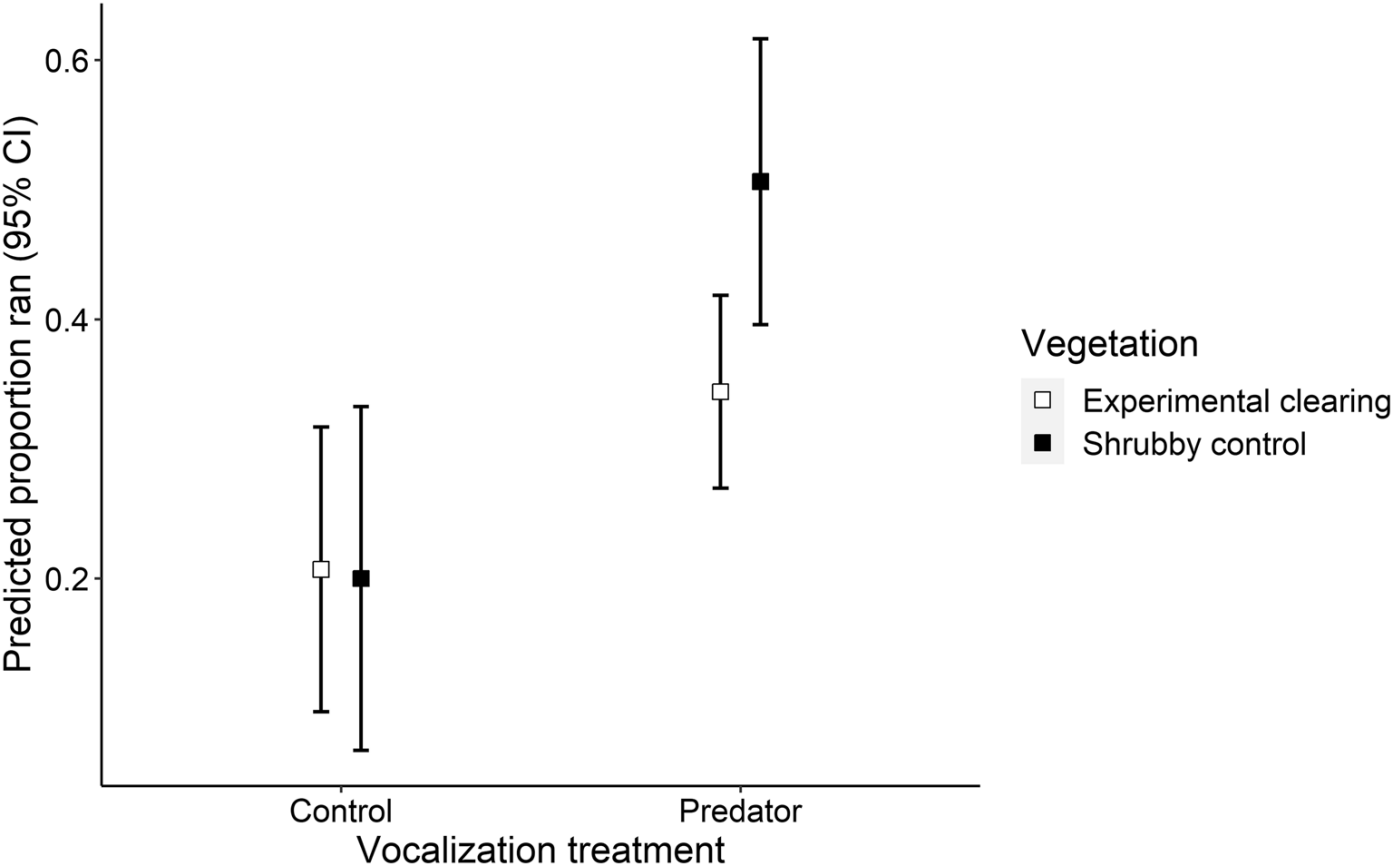
Predicted responses and 95% CIs from generalized linear models of ungulates running after hearing vocalizations of controls (birds) and large carnivores (dogs, hyenas and leopards) in experimentally cleared and shrubby control sites.

We evaluated the interactive proactive–reactive responses of all ungulates (pooled) by comparing vocalization and vegetation treatment categories (e.g., large carnivore vocalizations in experimentally cleared sites). We found ungulates had stronger reactive responses to large carnivore vocalizations in shrubby control sites than experimental clearings (Fig. 3). Ungulates (pooled) were 47% more likely to run after hearing a large carnivore vocalization in shrubby control sites than experimental clearings (Fig. 3; β = 1.37 ± 0.41 SE p < 0.001), demonstrating that ungulates perceived less fear of large carnivores on the sites they visited more often (clearings). Ungulates (pooled) that did not run were similarly vigilant in shrubby control and experimental clearings in response to hearing any large carnivore vocalization.

**Figure 4:**
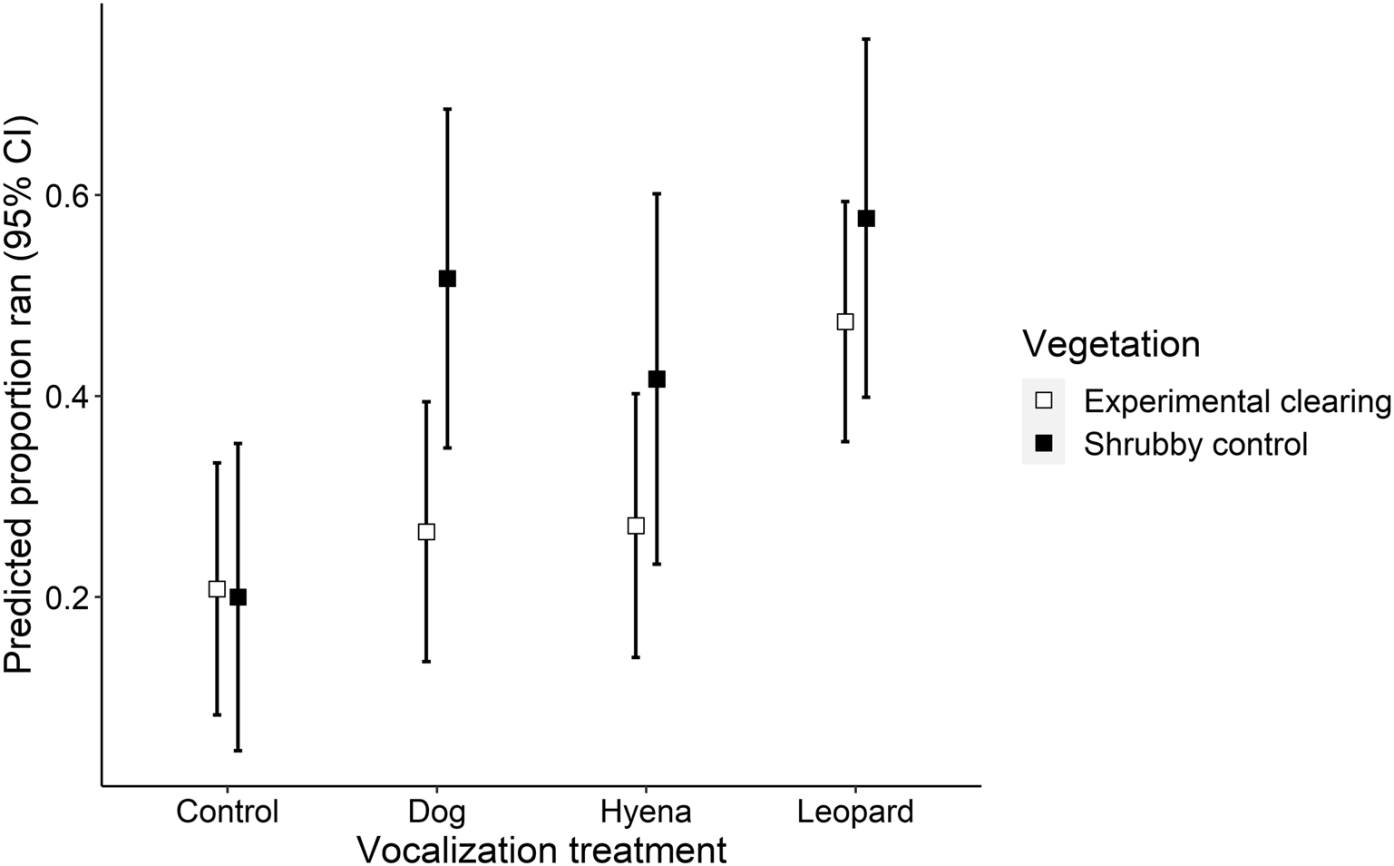
Predicted responses and 95% CIs from generalized linear models of ungulates running after hearing vocalizations of controls (birds), dogs, hyenas and leopards in experimentally cleared and shrubby control sites.

Providing more evidence that ungulates perceived shrubby control sites to be more dangerous, they were more likely to run from each carnivore vocalization in shrubby control sites than in experimental clearings. Ungulates (pooled) demonstrated an equally high likelihood of running in reaction to all types of large carnivore vocalizations in shrubby control sites (Fig. 4: dog, 52%; hyena, 42%; leopard 58%), whereas they were less reactive to dog and hyena vocalizations in experimental clearings (Fig. 4: dog, 27%; hyena, 27%; leopard, 48%). Ungulates ran significantly more in response to hearing leopards in both *Vegetation* types (clearings: β = 0.27 ± 0.08 SE p = 0.003; shrubby: β = 0.37 ± 0.11 SE p < 0.001) compared to control vocalizations in experimental clearings (Fig. 4). However, *Vegetation* type influenced the likelihood of running in response to the other 2 large carnivores. When compared with control vocalizations in experimental clearings, ungulates ran more from dog and hyena vocalizations at shrubby control sites (dog: β = 0.31 ± 0.11 SE p = 0.004; hyena: β = 0.21 ± 0.11 SE p = 0.067) (Fig. 4).

### Visitation responses to experimental clearings

**Figure 5:**
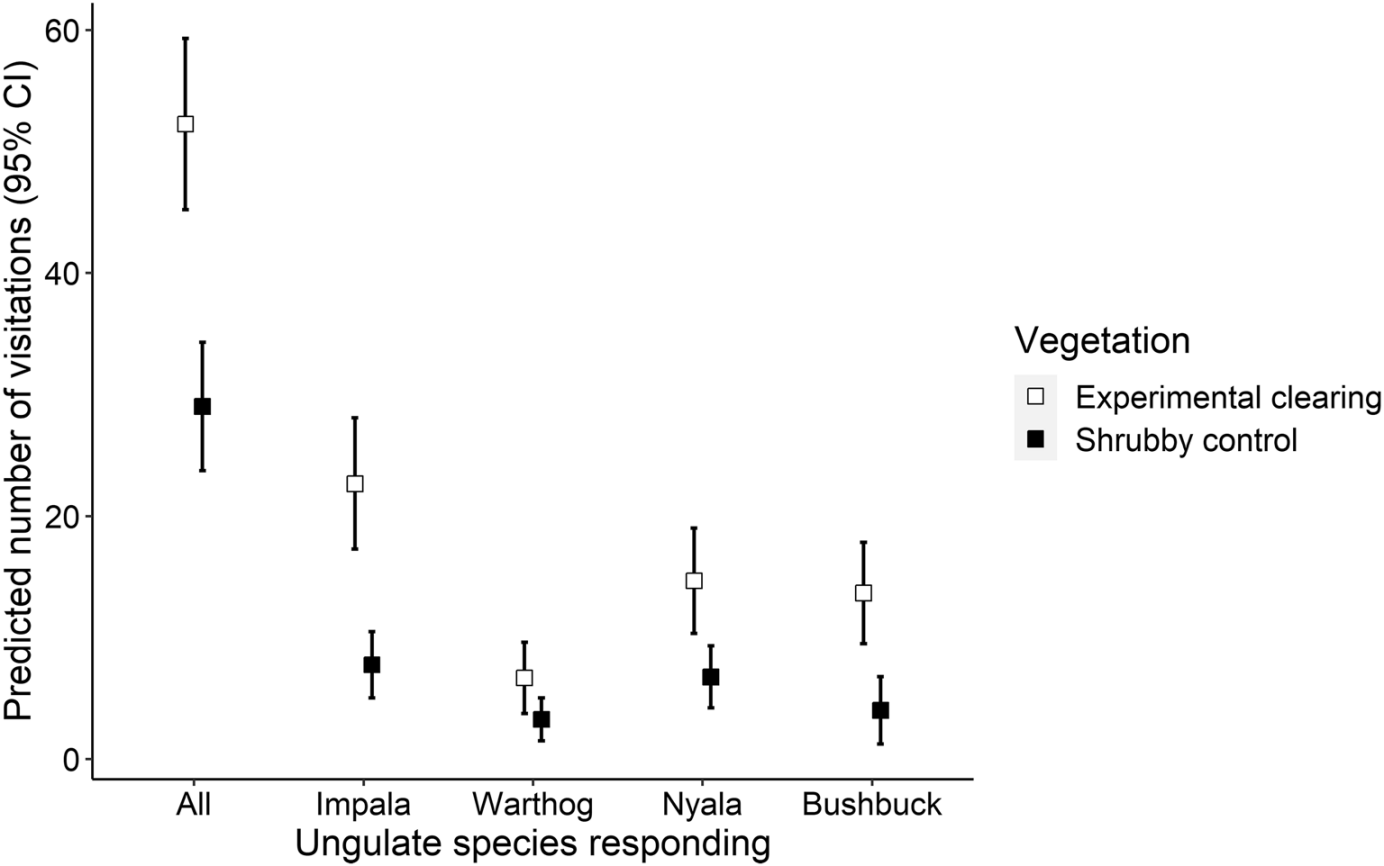
Predicted number of visitations and 95% CIs from generalized linear models of ungulates visiting experimentally cleared and shrubby control sites, considering all ungulates combined and the four most frequently detected species of ungulates.

We examined differences in the proactive visitation rates of all ungulates (pooled) and common ungulate species by *Vegetation* type (experimental clearings and shrubby controls) using glms. There were significantly more detections in experimental clearings than shrubby control sites considering all ungulates together (pooled). Ungulates were detected 2.4 times more frequently on average in experimental clearings than shrubby control sites (Fig. 5; clearings: n = 209 across 3 plots (excluding naturally open plot); shrubby: n = 116 across 4 plots; β = − 0.59 ± 0.12 SE p < 0.001). The most common ungulate species used experimental clearings significantly more often than shrubby control sites (Fig. 5). Impala were detected 2.3 times more frequently in experimental clearings, warthog 1.5 times, nyala 1.6 times, and bushbuck 5.1 times (impala: β = − 1.07 ± 0.22 SE p < 0.001; warthog: β = − 0.72 ± 0.36 SE p = 0.044; nyala: β = − 0.78 ± 0.24 SE p = 0.002; bushbuck: β = − 1.23 ± 0.39 SE p = 0.001).

**Figure 6:**
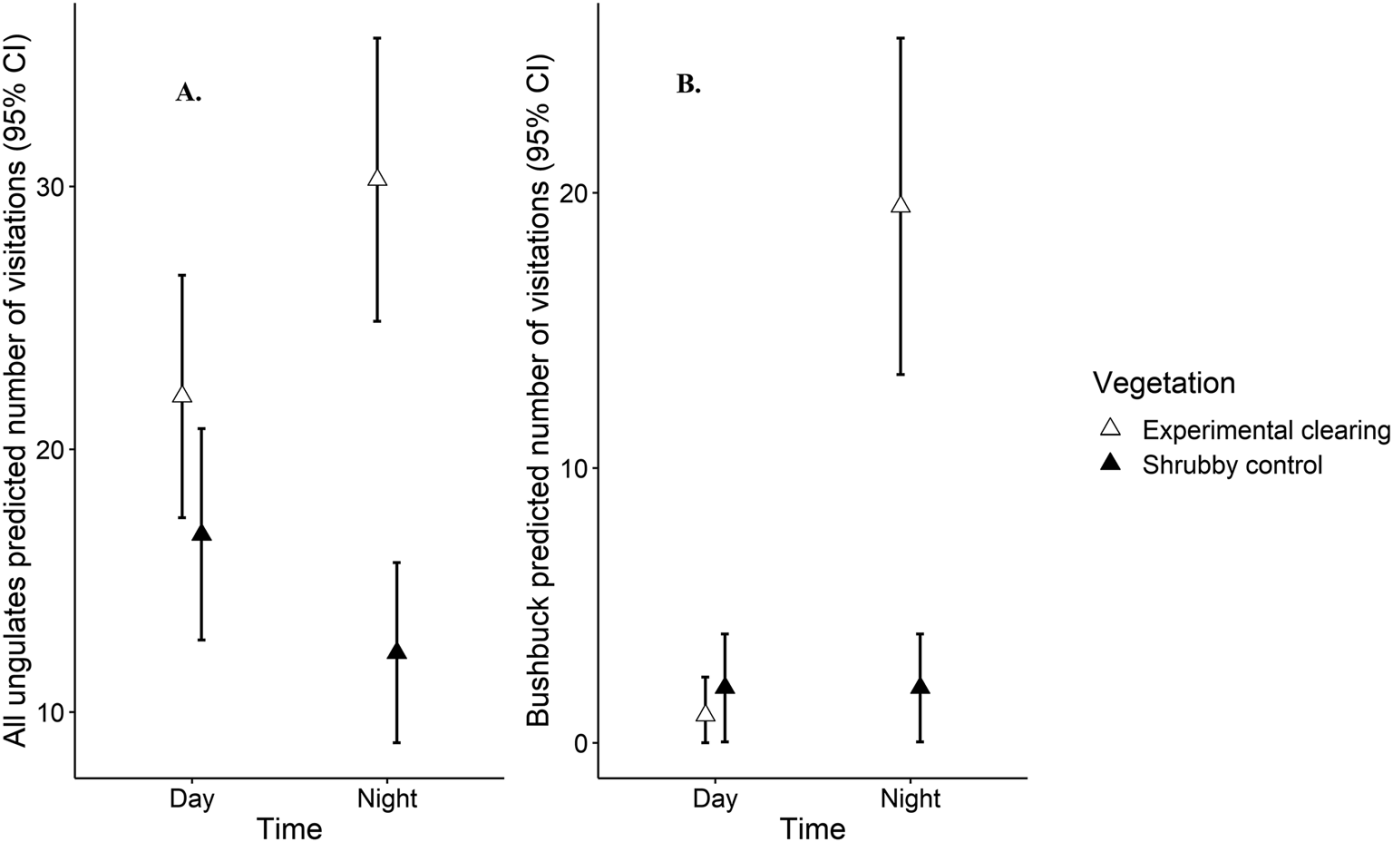
Predicted number of visitations and 95% CIs from generalized linear models of ungulates visiting experimentally cleared and shrubby control sites during the day or night, considering all ungulates combined (**A**) and bushbuck (**B**).

Consistent with the use of experimental clearings being a proactive response of ungulates to perceived predation risk, we found further differences in activity at night when large carnivores are more likely to be active. Pooled ungulate activity (i.e., visitation frequency) was greater at night (β = 0.32 ± 0.14 SE p = 0.023), but only in experimental clearings (Fig. 6A), resulting from a significant *Vegetation* (experimental clearings or shrubby controls) by *Time* (day or night) interaction (β = 0.63 ± 0.23 SE p = 0.007). All the common ungulate species were less likely to visit shrubby control sites (Fig. 5). However, considering each species separately, only bushbuck showed a significant response to *Time* and the interaction between *Time* and *Vegetation* (Fig. 6B; fewer detections during the day, β = − 2.97 ± 0.73 SE p < 0.001; proportionally more detections at night in experimental clearings, β = 2.97 ± 1.01 SE p = 0.003).

## Discussion

Manipulating both fear and shrub cover, we demonstrated interactive proactive–reactive responses, establishing a clear link between fear of large carnivores and ungulate habitat use. Ungulates visited experimental clearings more often than shrubby control sites, which is indicative of a proactive avoidance of shrubby areas (Fig. 5). Ungulates also showed heightened reactive responses to large carnivore vocalizations in shrubby areas (Figs. 3, 4), together demonstrating that ungulates perceived there to be less to fear from large carnivores in the sites they visited more often (clearings). This experimentally demonstrated interaction between ungulates’ proactive habitat use and reactive antipredator responses fills a critical gap in establishing that the fear large carnivores inspire in large ungulates can contribute to trophic cascades. Previous exclosure experiments aimed at linking fear of large carnivores to their habitat usage have established that differential habitat use by two of the species in our study, impala and bushbuck, can alter vegetation^5,13^. However, neither experiment demonstrated a direct linkage between fear and habitat use. Ford et al.^13^ conducted a vegetation thinning experiment demonstrating that impala select open areas over shrubby ones, and speculated, but did not directly test, that fear was the causal mechanism. Other vegetation thinning experiments have similarly demonstrated that impala and other ungulates select open areas^16,17^. Conversely, Atkins et al.^5^ manipulated fear in bushbuck by broadcasting leopard vocalizations but did not manipulate habitat characteristics. Given the experimental evidence that differential use of habitat by ungulates leads to alterations in ecosystem properties^5,13^, our experiment linking fear of large carnivores to ungulate habitat use provides compelling evidence that large carnivores can cause trophic cascades through fear alone. While we can not rule out the possibility that differences in habitat use were influenced by habitat quality, Ford et al.^13^ clearly established that impala’s avoidance of shrubby areas and selection of cleared areas could not be explained by forage quality or quantity.

Our experimental results clearly demonstrate that habitat characteristics affect the fear large ungulates manifest in response to large carnivores. Ungulates elevated fear response in shrubby control sites (Fig. 3) was likely a function of the increased structural complexity in shrubby habitats providing cover for large carnivores to hide, obscuring sightlines, and reducing the number of potential escape routes^13,55,56^. Large carnivores predominately hunt at night to reduce detection by their prey^28,29^, and the impediments to detecting large carnivores in shrubby vegetation are thus likely to be exacerbated at night. Accordingly, we predicted^13,21^ and found that ungulates, particularly the predominantly nocturnal bushbuck, increased visitations to experimental clearings at night (Fig. 6), corroborating our experimental demonstration that fear of large carnivores shapes large ungulate use of habitats.

The growing emphasis on developing a better understanding of the interactions among multiple prey and predators within large carnivore—ungulate systems^15,26,57^ is being driven, in part, by new methodologies to study behavior^10^. For example, using ABRs enabled us to test responses to playbacks during night and day on numerous species^38^. Nocturnal bushbuck, for example (Fig. 6B)^34^, would have likely been excluded in conventional diurnally conducted playback experiments [e.g.,^50^]. Consequently, the ABRs allowed us to uncover contrasts in anti-predator responses between diurnal and nocturnal species. For example, predominantly diurnal warthogs ran from the predator playbacks (Fig. 2A) while predominantly nocturnal bushbuck, which commonly freeze when they perceive predators^34^, significantly increased their vigilance rather than running (Fig. 2B). Moreover, our results confirm that a better understanding of diel variation in activity may be necessary as it likely plays an important role in mediating the influence of vegetation on large carnivore-ungulate interactions^58,59^.

With large carnivores declining in many environments^1,5^ while being reestablished in others^60,61^, it is critical that we understand how large ungulates vary their fear responses to different large carnivores^62^. In our experimental clearings, ungulates demonstrated a ‘hierarchy of fear’. As predicted, they responded more to leopard vocalizations than the vocalizations of the other two large carnivores (Fig. 4). In contrast, ungulates reacted more uniformly to all large carnivores in shrubby control sites (Fig. 4), likely corresponding with a reduction in the ability of ungulate prey to detect and evade any and all large carnivores in structurally complex shrubby vegetation^13,55,56^. Beyond illustrating the merit of testing responses to multiple large carnivores, we suggest our results also illustrate the value of collecting multiple behavioral measures (i.e., proactive and reactive) and examining mitigating factors (e.g., diel periods, prey species and predators) to generate a far more comprehensive understanding of how and when large carnivores can cause trophic cascades^3^.

Establishing whether fear of large carnivores contributes to causing trophic cascades is directly relevant to their conservation^3–5^. With woody vegetation encroaching on grassland systems across the globe^18^, we suggest our findings have important conservation implications. All ungulates appeared to avoid shrubby areas and increased their antipredator behaviors in response to all large carnivores when in shrubby areas. Accepting that ungulate avoidance of shrubby areas contributes to causing a trophic cascade, decreased browsing in shrubby areas may be expected to increase the growth and spread of woody vegetation^63,64^, leading to further avoidance, and thus creating a positive feedback loop. The increased avoidance of denser and more expansive shrubby areas could result in higher densities of ungulates in increasingly smaller open patches, potentially increasing competition among them, thereby leading to local declines in some large ungulates^20^.

Whereas, as our results experimentally demonstrate, fear mediates the avoidance of shrubby areas by large ungulates, other evidence indicates that smaller mammals show reductions in fear as shrub cover increases^65^. As shrub encroachment increases, these inter-specific differences in the perception of predation risk may exacerbate the ongoing patterns of global loss and replacement of large mammals with smaller ones^11,65^. More than the reduction of larger ungulates alone, the replacement of large consumers with smaller ones is likely to have important trophic consequences that reshape the structure and demographics of plant communities^2,11,66^.

## Methods

### Site description

Our manipulations were conducted in the low-lying savannas of northeastern Eswatini (formerly Swaziland) in the Mbuluzi Game Reserve (30 km^2^) and Mlawula Nature Reserve (165 km^2^). The vegetation here has distinct grass, shrub and tree layers^31^. The dominant trees were knobthorn (*Senegalia nigrescens*) and marula (*Sclerocarya birrea*), the dominant shrub was sicklebush (*Dichrostachys cinerea*), and the dominant grass species were red grass (*Themeda triandra*) and Guinea grass (*Panicum maximum*)^32^. Shrub cover, particularly sicklebush, increased on our site from 24% in 1998 to 44% in 2008^32,33^. The most common mid- to large-ungulates (≥ 15 kg) found on our sites included the impala, warthog, nyala and bushbuck^20^. Warthogs and impala are most active during the day and likely to become alert and flee when they perceive risk^34^. Both Nyala and bushbuck are more nocturnal and use a strategy of concealment, freezing before fleeing when they perceive risk^34^.

Large carnivores occurring in low densities on our sites included leopard and spotted hyena. We have also documented poachers with domestic dogs on our study site. Both carnivores and poachers with dogs are known to hunt and kill the common ungulates on our sites^35–37^.

### Experimental design

We used a bi-factorial experiment, manipulating both shrub cover and fear (the perceived presence of large carnivores), to test ungulates’ proactive and reactive antipredator responses. To experimentally test whether ungulates avoid areas with shrubby cover, we removed all sicklebush from designated areas covering at least 50 m × 50 m and compared ungulate visitation frequency with paired shrubby control sites during day and night. We monitored visitations with camera traps that were part of an Automated Behavioral Response system (ABR), comprised of an integrated motion-activated camera and speaker^38^. To experimentally test the reactive responses of ungulates to different large carnivores in the two habitats (experimental clearings and shrubby control sites), we exposed them to the vocalizations of several large carnivores (leopard, spotted hyena and dog) or controls (i.e., bird calls). Each ABR was programmed to broadcast all four playback treatments (leopard, hyena, dog and control) at set intervals (details below) thus providing a powerful sampling of all the individuals at that site^39^. Moreover, since the data collected by each ABR comprises a stand-alone playback experiment (complete with treatment and control vocalizations) use of multiple ABRs represents multiple replicates of the same experiment^38^. From videos recorded by the ABRs, we scored behaviors before and after the start of the vocalization and recorded whether ungulates ran, a definitive behavioral measure of fear^40^. As an ancillary measure, we examined changes in vigilance behaviors of ungulates that did not run. We compared the responses of common ungulate species to the different large carnivores. Finally, to experimentally test for a proactive–reactive interaction, we compared responses to the vocalizations of any large carnivore, and each separately, in experimentally cleared and shrubby control sites to determine if ungulates reacted with less fear in frequently used habitat. All research was conducted under the University of Florida IACUC (Protocol #201810212) and with the permission of Mbuluzi Game Reserve and Mlawula Nature Reserve.

### Experimental clearings

To manipulate shrub cover, we removed all sicklebush from 3, 50 m × 50 m areas between November 2017 and March 2018. The average stem circumference growth of sicklebush is 0.6 mm per month, with little regrowth prior to conducting our experiments in June and July 2018^41^. We examined proactive responses using experimentally cleared sites (n = 3, shrub cover = 7–17%) matched with a non-manipulated shrubby control sites (n = 3, shrub cover = 41–87%) that were > 500 m apart and within daily movements of most of the common ungulates in our study^42^. In an effort to limit an ungulate’s exposure to only one matched site (i.e., independence of matched sites), we placed matched sites > 2 km apart, outside of the seasonal ranges of most common ungulates in our study^42^. In testing reactive responses to hearing large carnivore vocalizations (see below), we increased our sample sizes by further selecting a naturally open site that we matched with an additional shrubby control site (shrub cover = 52%). The naturally open site was similar to the other experimentally cleared sites with 20% shrub cover. We estimated shrub cover using the line intercept method^43^.

### Automated behavioral response (ABR) system

At each site, we strapped a camera trap to a tree at 1.5 m height, set the focal point of the camera to 8 m directly in front of the camera, and clipped vegetation within a 15 m radius of the front of the camera to reduce false triggers, and ensure 100% detection at 8 m (Supplementary Videos S1 and S2), 15 m being the detection range of the camera’s motion sensor. This created a consistent visual arena to ensure no variation in detection between experimentally cleared and shrubby control sites. We placed a speaker connected to the camera 0.5 m above the camera. Following a well-established protocol used in previous ABR experiments, we set the camera to begin recording once motion was detected, and after a 3-s. delay^38,39^ the speaker broadcast a 10-s. vocalization. We set the camera to record 30-s. videos, but were restricted (by the camera’s design) to recording 20-s. videos at night. We programmed the ABRs with 8 exemplars each of the vocalizations of each large carnivore and the bird calls (African hoopoes [*Upupa Africana*] during the day and fiery-necked nightjars [*Caprimulgus pectoralis*] at night [Supplementary Methods]^44^). We used the camera’s classification of day or night (via light sensor) for our analyses and deployed ABRs for 38 consecutive 24-h. periods at the 6 original sites and 30 consecutive 24-h. periods at the additional naturally open and shrubby control sites.

### Scoring behavior

For each video we recorded site, date, time (30-s. day or 20-s. night video), species, vocalization treatment, and whether the video was a first or repeat exposure. We considered videos of a given species to be independent first exposure videos if > 60 minutes had elapsed since the last time that species heard the same vocalization at that site, which is a conservative classification^45,46^. We scored whether the animal *Ran* after the vocalization began because it is a definitive behavioral measure of fear^40^. As a supporting measure, for animals that did not run, we calculated the proportion of time vigilant (head up) before and after the start of the vocalization, the difference between the two being ‘Vigilance’. These types of vigilance behaviors, along with fleeing, can be costly anti-predator responses that reduce foraging opportunities^47,48^. We scored the behavior of the ungulates(s) (Supplementary Methods) in the frame before and after the start of the vocalization (Supplementary Videos S1 and S2) based on behavior descriptions used in previous studies^49–51^. We used the broad classifications head up, head down, stand, walk and run as these classifications most unambiguously identify the behavior state (Supplementary Methods, Supplementary Table S1).

### Statistical analyses

#### Reactive responses to experimental predator vocalizations

We evaluated the reactive responses of ungulates to large carnivore vocalizations in two ways: (1) we compared the reactions to the vocalization of each large carnivore species considered separately vs. the controls (*Large carnivore species* vs. *control* hereafter); and (2) we compared the reactions to hearing any large carnivore (large carnivores pooled) vs. the controls (*Large carnivore* vs. *control* hereafter).

We examined antipredator behaviors primarily as running or not running after vocalizations, and secondarily as vigilance behaviors. For both, we included only first exposure videos (i.e., independent samples) in our analysis. We evaluated difference in ungulates run response by developing glms with a binomial distribution (logit link) using *Ran* as the binary response variable (Supplementary Table S2). To assess the *Vigilance* response variable, we transformed (*x*^3^) the skewed data, and developed glms with a Gaussian distribution (Supplementary Table S2). We performed all glms using the base package in R platform *v* 3.4.1 (R Core Development Team 2016). We evaluated categorical responses with a Wald test (p < 0.05) and examination of 95% CIs of beta estimates. With 3 pairs of matched sites (experimentally cleared and shrubby control), using a mixed model to account for potential variation among pairs would likely lead to imprecise estimates of random effect; however, a practical alternative is to treat the pairs as a fixed effect^52,53^. Accordingly, we considered matched sites as a fixed effect and compared it to an intercept only model in a single variable model. Finding no explanatory value in this variable, we excluded this variable from further analyses (*Ran*, likelihood ratio statistic = 3.60, *p* = 0.308; *Vigilance*, likelihood ratio statistic = 1.95, *p* = 0.583).

We determined if ungulates (pooled) ran more frequently or were more vigilant after each large carnivore species’ vocalizations than control vocalizations by modeling *Ran* and *Vigilance* separately with the fixed effect *Large carnivore species* vs. *control* with control vocalizations as the reference category (Supplementary Table S2). To determine if ungulates varied in their responses to different large carnivores, we modeled *Ran* and *Vigilance* separately, setting the potentially more lethal leopard^26,30^ as the reference category and comparing it to responses to hyenas and dogs. Next, we evaluated variation in responses between common ungulate species by comparing species with sufficient samples (> 30 first exposures)^54^. We modeled the *Ran* and *Vigilance* responses of each species with the fixed effect *Large carnivore vs. control*.

#### Interactive proactive–reactive responses to the bi-factorial experiment

To test the prediction that ungulates were more afraid of large carnivores in shrubby control sites, we compared *Ran* and *Vigilance* responses to large carnivore vocalizations (large carnivores pooled) in shrubby control sites (excluding samples from the naturally open site) to *Ran* and *Vigilance* responses to (1) large carnivore vocalizations in experimentally cleared sites, (2) control vocalizations in shrubby control sites, and (3) control vocalizations in experimentally cleared sites. To determine if ungulates’ responses varied by predator-specific vocalizations between experimentally cleared and shrubby control sites, we compared *Ran* responses on cleared sites with control vocalization to (1) control vocalizations in shrubby control sites, (2) dog vocalizations in experimentally cleared sites, (3) dog vocalizations in shrubby control sites, (4) hyena vocalizations in experimentally cleared sites, (5) hyena vocalizations in shrubby control sites, (6) leopard vocalizations in experimentally cleared sites, and (7) leopard vocalizations in shrubby control sites. We evaluated the categorical responses with a Wald test (*p* < 0.05) and examination of 95% CI of beta estimates.

### Visitation responses to experimental clearings

To test the potential for proactive responses of ungulates to the shrub removal manipulation, we evaluated differences in site visitations. We developed generalized linear models (glms), fitting visitations (number of visitations per site) to a Poisson distribution (log link) as the response variable and *Vegetation* (experimental clearings or shrubby controls) as the fixed effect (Supplementary Table S2). In this analysis, we included first exposure camera detections (i.e., independent samples) and to isolate the effect of shrub clearing, we only used open sites that were experimentally cleared. To further test if visitation rates varied temporally as a function of vegetation cover^21^, we developed interactive models with *Vegetation* and *Time* (day or night) as main effects, with the addition of an interaction effect between them (Supplementary Table S2). We modeled the response of all ungulates pooled and of individual species with sufficient first exposure samples (> 30)^54^. We evaluated model parameters with a Wald test and considered them to be significant at *p* < 0.05. As above, we consider each pair of matched sites as a categorical fixed effect and compared a model with a fixed effect for matched sites and an intercept only model. Finding no support for variation among matched sites, we excluded this variable from further analyses (likelihood ratio statistic = 3.34, *p* = 0.342).

## Supplementary Information

Supplementary Information 1.

Supplementary Video 1.

Supplementary Video 2.

## Data Availability

Data will be made available through Dryad.
