# Publisher Correction: Fear of large carnivores is tied to ungulate habitat use: evidence from a bifactorial experiment

**DOI:** 10.1038/s41598-021-93822-4

**Published:** 2021-07-06

**Authors:** Haley K. Epperly, Michael Clinchy, Liana Y. Zanette, Robert A. McCleery

**Affiliations:** 1 Department of Wildlife Ecology and Conservation, School of Natural Resources and the Environment, University of Florida, Gainesville, FL 32611 USA; 2 Department of Biology, Western University, London, ON N6A 5B7 Canada; 3 University of Florida, 110 Newins‑Ziegler Hall, PO Box 110430, Gainesville, FL 32611‑0430 USA

Correction to: *Scientific Reports* https://doi.org/10.1038/s41598-021-92469-5, published online 21 June 2021

The original version of this Article contained an error in the spelling of the author Robert A. McCleery which was incorrectly given as Robert A. McCeery. The original Article has been corrected.

