# Supplementary material for: Fear of large carnivores is tied to ungulate habitat use: evidence from a bifactorial experiment
Source: Sci Rep. 2021 Jun 21;11:12979. doi: 10.1038/s41598-021-92469-5 (PMC8217516; doi:10.1038/s41598-021-92469-5)
Supplement: Supplementary file 1 — Supplementary Information 1. [file 41598_2021_92469_MOESM1_ESM.docx]

**Supplementary Information.** Haley K. Epperly, Michael Clinchy, Liana Y. Zanette, and Robert A. McCleery. 2021.

Fear of large carnivores is tied to ungulate habitat use: evidence from a bifactorial experiment.

**Methods**

**Creating and Randomizing Predator and Control Vocalizations**

We created 8 different 10-second audio recordings for each vocalization type to simulate realistic variation in animal vocalizations and to account for potential variation in perceived threat within vocalization treatments (e.g. male vs female vocalization). We collected original audio recordings from a variety of sources including the Xeno-Canto website (www.xeno-canto.org), YouTube (www.youtube.com) and collaborating scientists. We cleaned the audio files by removing background noises using Audacity (Audacity 2.2.2). We set the average volume of each 10-second vocalization to 80 decibels at 1m using the app Decibel X (SkyPaw Co. Ltd.) and made minor volume adjustments based on how the vocalizations sounded in the field to ensure that the volume sounded consistent across all treatments.

If the Automated Behavioral Response system (ABR) was triggered, we programmed each vocalization treatment to be broadcast during a 15-minute block each hour, with the first vocalization’s block beginning at the start of the hour. During the 15-minute blocks, we programed the ABR to randomly select and play 1 of 8 vocalizations each time the camera was triggered. For example, if the camera was triggered between 1:00-1:15am, the ABR system randomly played 1 of 8 unique leopard vocalizations, between 1:15-1:30am the ABR system randomly played 1 of 8 unique dog vocalizations. When an animal triggered the ABR system multiple times within a 15-minute block, the same vocalization type was played. To control for order effects, we randomized the order in which the 4 treatments were played in each hour, throughout the 24-hour day, and additionally compiled 2 playlists with different random orders. We set 4 ABRs to follow the order from one list and the other 4 ABRs to follow the order from the second list. We set coupled experimentally cleared and shrubby control site ABRs to use the same list.

**Additional Ungulate Behavior Scoring Information**

We first scored the videos blind to the vocalization treatment, without audio, to reduce bias. To avoid pseudoreplication, we used the video as the unit of analysis rather than the individual ungulate(s) within the video (Dröge et al. 2017). For videos with more than one ungulate, we classified the sample as ran if ≥ 50% ran and used the median duration vigilant across all individuals. We classified all videos using Solomon Coder software (Solomon Coder version beta 17.03.22). After we classified the behavior of the ungulate(s), we watched the videos with audio and recorded which vocalization treatment had been broadcast.

**Table S1**. Ethogram used to score behaviors of ungulates from camera trap videos recorded at Mbuluzi Game Reserve and Mlawula Nature Reserve, Eswatini from June-July 2018. Ungulates were exposed to a 10-second treatment call from a speaker and video recorded for 30-seconds during the day and 20-seconds at night.

| Movement | | |
| --- | --- | --- |
| Category | Type | Description |
| Ran | Continuous (seconds) | Animal that took at least three leaps while moving at a quick pace with both front legs moving in conjunction and both back legs moving in conjunction |
| Walk | Continuous (seconds) | Animal moving at a slow pace and alternating  movement of the right and left legs |
| Stand | Continuous (seconds) | Animal with all four hooves touching the ground |
| Attention | | |
| Category | Type | Description |
| Alert | Continuous (seconds) | Animal with head facing up (head at 90 degree angle or greater from ground) |
| Not alert | Continuous (seconds) | Animal with head facing down (head at less than 90 degree angle from ground) |

**Table S2.** Name and type of response variables (Y) and fixed effects (X) and their respective data type, number, measurement descriptions and units used to examine the relationship between visitation, vigilance and run behaviors and ungulate species, large carnivore vocalizations and vegetation treatments.

| Variable | X/Y | Type (#) | Measurement |
| --- | --- | --- | --- |
| Ran | Y | Categorical (2) | Ran after or did not run after start of vocalization |
| Visitation | Y | Discrete | Number of first exposure videos at each site |
| Vigilance | Y | Continuous | Change in proportion of time vigilant (head up) before and after start of vocalization |
| Large carnivore species vs. control | X | Categorical (4) | Dog, hyena, leopard and pooled controls (nightjar and hoopoe) |
| Large carnivore vs. control | X | Categorical (2) | Pooled large carnivores (dog, hyena and leopard) and pooled controls (nightjar and hoopoe) |
| Species | X | Categorical (4) | Impala, warthog, nyala and bushbuck |
| Time | X | Categorical (2) | Day (30-second video recorded) or night (20-second video recorded) |
| Vegetation | X | Categorical (2) | Experimentally cleared or shrubby control sites |
